# Glutamatergic Dysbalance and Oxidative Stress in *In Vivo* and *In Vitro* Models of Psychosis Based on Chronic NMDA Receptor Antagonism

**DOI:** 10.1371/journal.pone.0059395

**Published:** 2013-07-15

**Authors:** Just Genius, Johanna Geiger, Anna-Lena Dölzer, Jens Benninghoff, Ina Giegling, Annette M. Hartmann, Hans-Jürgen Möller, Dan Rujescu

**Affiliations:** 1 Department of Psychiatry, Ludwig-Maximilians University of Munich, Munich, Germany; 2 Department of Psychiatry, University of Essen, Essen, Germany; 3 Department of Psychiatry, University of Halle, Halle, Germany; Chiba University Center for Forensic Mental Health, Japan

## Abstract

**Background:**

The psychotomimetic effects of N-methyl-D-aspartate (NMDA) receptor antagonists in healthy humans and their tendency to aggravate psychotic symptoms in schizophrenic patients have promoted the notion of altered glutamatergic neurotransmission in the pathogenesis of schizophrenia.

**Methods:**

The NMDA-receptor antagonist MK-801 was chronically administered to rats (0.02 mg/kg intraperitoneally for 14 days). In one subgroup the antipsychotic haloperidol (1 mg/kg) was employed as a rescue therapy. Glutamate distribution and 3-NT (3-nitrotyrosine) as a marker of oxidative stress were assessed by immunohistochemistry in tissue sections. In parallel, the effects of MK-801 and haloperidol were investigated in primary embryonal hippocampal cell cultures from rats.

**Results:**

Chronic NMDA-R antagonism led to a marked increase of intracellular glutamate in the hippocampus (126.1 +/− 10.4% S.E.M of control; p = 0.037), while 3-NT staining intensity remained unaltered. No differences were observed in extrahippocampal brain regions. Essentially these findings could be reproduced *in vitro*.

**Conclusion:**

The combined *in vivo* and *in vitro* strategy allowed us to assess the implications of disturbed glutamate metabolism for the occurrence of oxidative stress and to investigate the effects of antipsychotics. Our data suggest that oxidative stress plays a minor role in this model than previously suggested. The same applies to apoptosis. Moreover, the effect of haloperidol seems to be mediated through yet unidentified mechanisms, unrelated to D2-antagonism. These convergent lines of evidence indicate that further research should be focused on the glutamatergic system and that our animal model may provide a tool to explore the biology of schizophrenia.

## Introduction

N-methyl-D-aspartate (NMDA) receptor antagonists such as phencyclidine (PCP) and ketamine can elicit psychotomimetic effects in healthy humans and exacerbate psychotic symptoms in schizophrenic patients [1]–[5]. This observation promoted the conception of schizophrenia as a condition of an abnormal balance between glutamatergic neurotransmission and other neurotransmitter systems. Based on these findings a pharmacological model based on NMDA-R antagonism was developed, which is characterized by several remarkable parallels with genuine schizophrenia [6]–[13]. Currently, this model becomes increasingly accepted as a tool for the study of this condition. Emulating the chronic nature of the supposed impaired glutamate metabolism in schizophrenia, we followed a strategy of chronic, low-dose application of MK-801, a highly selective uncompetitive NMDA receptor antagonist, which binds to the PCP-binding site of the ion channel. The doses were selected in a range far below those required for the induction of anesthesia or acute behavioral effects but high enough to induce reproducible effects on gene expression patterns, electrophysiological measures and structural alterations paralleling those in schizophrenia: In an earlier report we were able to demonstrate working and declarative memory deficits resembling of those reported in schizophrenic patients [14] and a selective loss of parvalbumin-positive GABAergic interneurons. This neuronal population is suggested to facilitate working memory storage and retrieval through their gamma band oscillatory activity [15]. It was also shown that NMDA receptor antagonism may result in an altered NMDA receptor subunit expression pattern [16].

We were thus able to reproduce some of the most relevant and disturbing symptoms of schizophrenia including cognitive impairment, which –from our point of view- were insufficiently modeled in conventional approaches, e.g. based on interference with the dopaminergic system.

**Figure 1:**
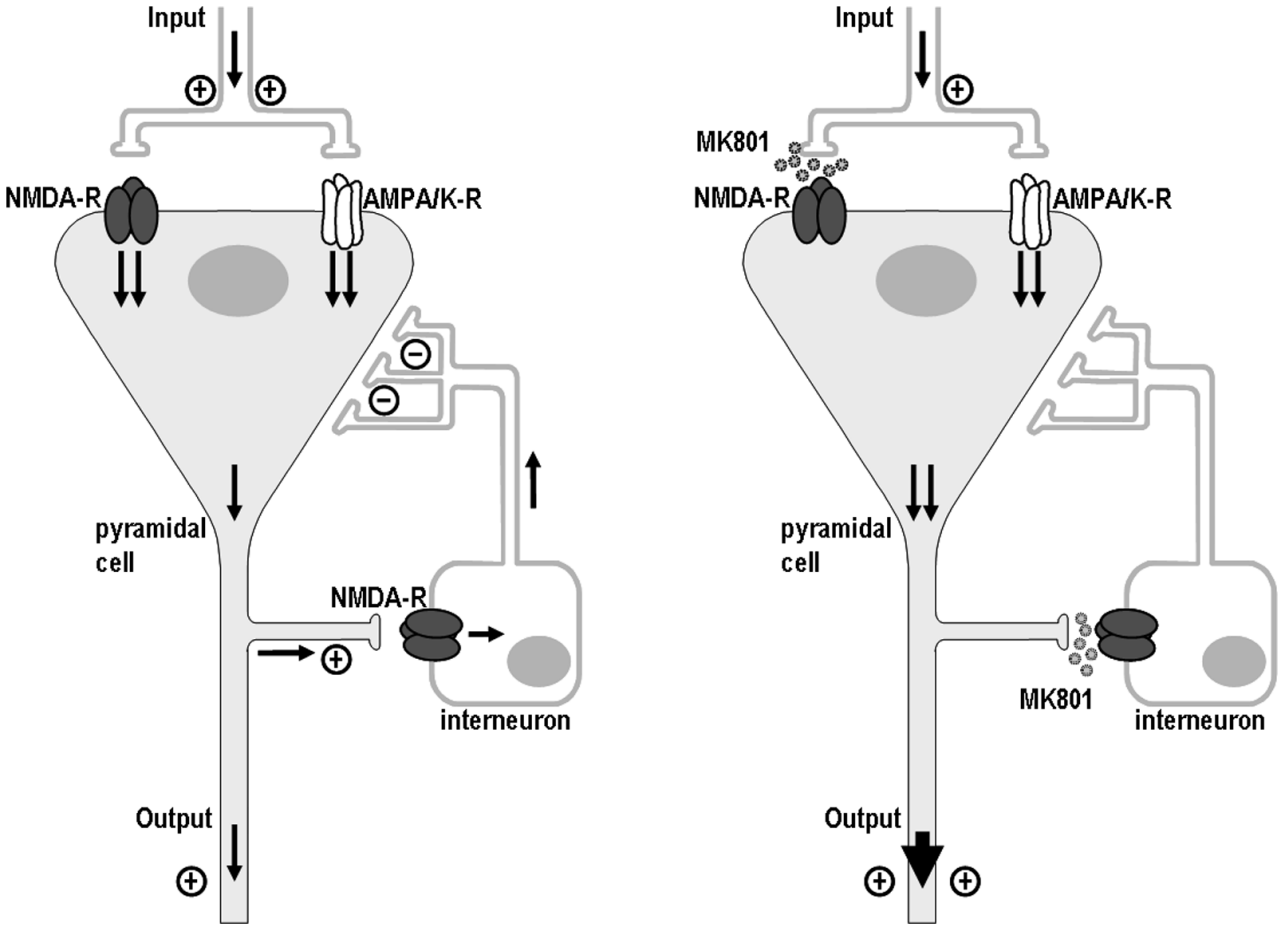
Model of the local neuronal circuit disinhibition elicited by MK-801. The GABAergic interneuron (IN) receives input from the pyramidal cell (PC) thereby exerting an inhibitory control by recurrent projections to the PC. In presence of the NMDA receptor antagonist MK-801, this local feedback inhibition becomes disrupted, whereas the excitatory input is sustained via non-NMDA (AMPA/kainate) receptors, which do not respond to MK-801. Due to this imbalance, the total excitatory output will be enhanced. (GABA, γ-aminobutyric acid; NMDA, N-methyl-D-aspartate).

We hypothesized, that chronic NMDA-R antagonism may lead to a profound dysregulation of the glutamatergic system (Fig. 1). Secondly, we would expect that this deranged glutamate metabolism may elicit an abnormal production of reactive oxygen intermediates (ROI) and thus result in excitotoxic neurodegeneration [17]–[20]. Indeed, accumulating data from previous studies implicate oxidative stress as one candidate mechanism for the pathogenesis of schizophrenia [17], [18]–[22]. A recent report has illustrated, that the loss of GABAergic interneurons induced by an NMDA-R antagonist (ketamine) is mediated through an enhanced generation of superoxide [23].

Previous studies have already (albeit inconsistently) shown elevated biomarkers of oxidative stress or altered concentrations of enzymatic or low-molecular antioxidant systems in body fluids or brains from schizophrenic patients [21], [24], [25]. Chronic oxidative stress may affect the gene expression pattern and may directly affect neuronal function and structural integrity. Our study was designed to model the same pattern of disturbed glutamate neurotransmission [26], [27] and subsequent oxidative stress. Identifying such relationships would add further weight to the validity of our animal model and may contribute to a better understanding of the mechanisms underlying psychotic behavior. Besides, the development of novel pharmacological approaches will be vitally dependent on the establishment of suitable animal or *in vitro* models.

## Materials and Methods

### Animal treatment

Male Long Evans rats (Janvier Breeding Centre, Le Genest Saint Isle, France) at the transition from puberty to adolescence (n = 48; age 35+/−1 days; initial weight 121–148 g) were matched according to body weight and housed in groups of four in atmospherically controlled cages, with a 12/12 h light/dark cycle, and food and water provided *ad libitum*. After an adaptation period of 7 days they received daily intraperitoneal (i.p.) injections (2 ml/kg body weight, 0.9% saline as vehicle) of either 0.02 mg/kg (+)-MK-801 maleate [(5R,10S)-(+)-5-methyl-10,11-dihydro-5I-dibenzo[a,b]cyclohepten-5,10-imine, dizocilpine, Sigma, Taufkirchen, Germany)] (n = 12) throughout the entire treatment period of 3 weeks or 1 mg/kg haloperidol in the 3^rd^ week after application of saline for 2 weeks (n = 11; one animal was killed in an accident). A further group (n = 12) received a combination of both agents (MK-801 throughout the entire experiment and an additional haloperidol “rescue” in the 3^rd^ week). The control group (n = 12) received a daily i.p. injection of 0.9% saline for 3 weeks. Drugs were applied during the light phase. While under deep CO2 anesthesia, rats were sacrificed by decapitation 24 h following the last drug administration. The right hemispheres (n = 47) were processed for immunohistochemistry.

All manipulations were performed in strict accordance with the current versions of the US and German Law for the Protection of Animals (approval ID: 209.1/211-2531-78/03 Regierung von Oberbayern Maximilianstr. 39 80538 Munich, Germany).

### Tissue section preparation

For immunostainings of intracellular glutamate and 3-nitrotyrosine, the hemispheres were fixed in 4% ice-cold para-formaldehyde (pH 6.4). After rinsing with PBS the hemispheres were cryoprotected by ascending concentrations of sucrose (15% in PBS for 12 h, 30% in PBS for 24 h at 4°C), embedded in TissueTek (Sakara, Torrance, CA, USA) and subsequently stored at –80°C. Coronal 17 µm cryosections were prepared at −14°C on a Leica cryostat (1720 digital, Leica, Bensheim, Germany) and mounted on Superfrost Plus slides (Erie Scientific Company, Portsmouth, NH, USA). After a 16 h drying period sections were stored at –80°C.

### Immunohistochemistry for L-glutamate and 3-nitrotyrosine

Antibody penetration and unmasking of epitopes was enhanced by microwave 3 times for 7 min each in a PBS-citrate buffer (pH = 6) supplemented with 0.05% Tween-20 at 750 W. The staining protocol and subsequent analysis were performed by an investigator blinded to the treatment status. Staining started with a blocking step (4% normal goat serum in PBS with 0,5% Triton-X100 for 1 h). Unspecific peroxidase activity was quenched by 10 min of incubation with 0.75% H_2_O_2_. Slides were incubated with monoclonal primary antibodies from rabbit directed against L-glutamate (1∶250) or 3-nitrotyrosine (1∶1000), (both from Chemicon, Temecula, USA) at 4°C over-night. Incubation with secondary biotin-conjugated goat anti-rabbit IgG antibodies (Dianova, Hamburg, Germany) was performed for 30 min at RT, followed by a further incubation step with horseradish-peroxidase-labeled streptavidin (15 min). Between each step, slides were rinsed in PBS for 2×20 min. The staining procedure was completed by an exactly timed incubation with a diaminobenzidine/NADPH detection solution and embedding in MobiGlow mounting solution (Mobitec, Göttingen, Germany). Negative controls, in which primary antibodies were omitted, revealed no reaction.

At least 8 sections per animal covering the dorsofrontal extension of the hippocampal formation between plane 47 (interaural 7.28 mm and Bregma −1.72 mm) and plane 87 (interaural 2.52 mm and Bregma −6.48 mm) according to Paxinos&Watson [28], were chosen and images were acquired at transmitted light with a digital camera (ProgRes C10+, Jenoptik, Jena, Germany) adapted to a Zeiss Axioplan 2 microscope (Carl Zeiss, Jena, Germany).

For quantification different regions of interest (ROI) within and outside the hippocampal formation were defined by two independent raters, quantified with the open-source ImageJ version 1.3.4 program (NIH, Bethesda, USA http://rsb.info.nih.gov/ij) and normalized to ROI placed in the molecular layer of the dentate gyrus.

### Hippocampal embryonic cell culture

Pregnant Long Evans rats (Janvier Breeding Centre, Le Genest Saint Isle, France) were killed by decapitation in deep CO_2_ anesthesia. The embryos (embryonic day 17/18) were rapidly micro-dissected for isolation of the hippocampi. These were dissociated by mechanical homogenization in a Hank`s balanced salt solution (HBSS) without Ca^2+^ and Mg^2+^ buffered with 10 mM HEPES at pH 7.4 and supplemented with 1 mM sodium pyruvate and 4% bovine serum albumin. For further dissociation, tissue was incubated with HBSS solution containing 2 mg/ml papain and 1000 kU/ml DNAse I. Debris was removed by two steps of centrifugation at 800 g for 15 min each and resuspension of the resulting pellet by gentle trituration. The live (dye-excluding) purified cells were counted in a hematocytometer, plated at a density of 0.8×10^5^ cells/cm^2^ and cultivated in a defined medium (Neurobasal with antioxidant-free B27 supplement, 0.5 mM glutamine, 50 μg/ml gentamycine, GIBCO BRL, Life Technologies Ltd, Paisley, UK) on L-ornithine-coated tissue culture dishes (Nalge Nunc International, Rochester, NY, USA) at 95% air and 5% CO_2_ in a humidified incubator. Unattached cells and debris were aspirated after 4 h. Twice a week one half of the medium volume was replaced. Experiments were performed on 9–11 DIV (days *in vitro*). Cell culture quality was routinely assessed by viability analyses, morphological parameters and immunostained for neuronal and glial cell markers. Glial cells identified by GFAP immunofluorescence represented <1% of the total cell population, while >99% of the cells expressed NeuN (neuronal nuclear protein) and β-3-tubulin (TUJ-1) as neuronal markers. In incubation experiments, Lactate dehydrogenase (LDH) efflux into the cell culture supernatant was used as a cumulative marker to determine cytotoxicity (CytoTox 96, Promega, Madison, WI, USA). Glutamate was measured by an enzymatic assay (Amplex Red™ Glutamate assay kit, Molecular Probes, Eugene, Oregon, USA). Protein concentration was determined by the Bradford assay (Biorad, Munich, Germany).

### Chemiluminescent determination of superoxide anions in hippocampal cells

Cells after 5–7 DIV (days *in vitro*) were harvested mechanically, dissociated and gently centrifuged. The resulting cell pellet was resuspended in Neurobasal medium. Aliquots of the cell suspension were transferred into the test tube and allowed to settle on the bottom to minimize light scattering. After addition of lucigenin (bis-N-methylacridiniumnitrate, 50 µM final concentration) the tube was transferred to a luminometer (LB9507, Berthold Technologies, Bad Wildbad, Germany) for further 15 minutes further to achieve reagent-uptake in the dark. The basic light output and the reaction towards pharmacological interventions were continuously recorded by measuring the light quantum yield at 2 sec intervals. Background chemiluminescence (CL) was subtracted. The specificity of CL for stimulated O_2_^.−^ release was verified by adding superoxide dismutase (SOD), the cell-permeable SOD mimic MnTBAP (manganese[III]tetrakis[4-benzoic acid]porphyrin), or the low molecular weight O_2_^−^ scavenger tiron (4,5-dihydroxy-1,3-benzene-disulfonic acid).

### Statistical analysis

If not otherwise specified, data were analyzed with the SPSS software version 12.0 (SPSS Inc., Chicago, IL, USA). For normally distributed data the Student`s t-test was performed if not otherwise specified. Due to nonparametric distribution of the immunohistochemical results, the two-tailed Mann-Whitney-U test was chosen to estimate differences for these data, which are expressed as means +/− S.E.M. All other data are expressed as means +/− SD. To assess relationships between markers Spearman’s correlation coefficient was calculated using linear regression. P values of <0.05 were considered as statistically significant.

## Results

### Treatment effects on hippocampal glutamate distribution and glutamate-related genes in the hippocampal formation in the animal model

**Figure 2:**
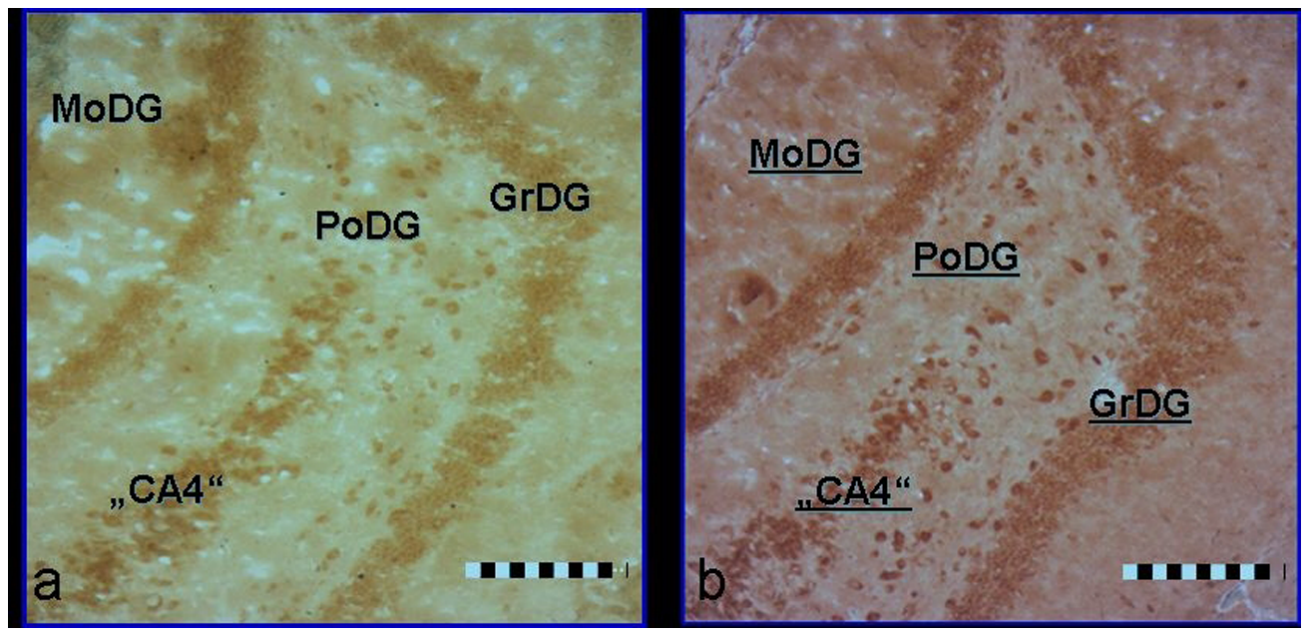
Immunohistochemical localization of glutamate(2a) and 3-nitrotyrosine (2b) in the hippocampal formation. Representative photomicrographs (scale bar = 200 µM). GrDG granular cell layer of dentate gyrus; MoDG molecular layer of dentate gyrus; PoDG polymorphic layer of dentate gyrus; “CA4” terminal portion of the hippocampal pyramidal cell layer.

**Figure 3:**
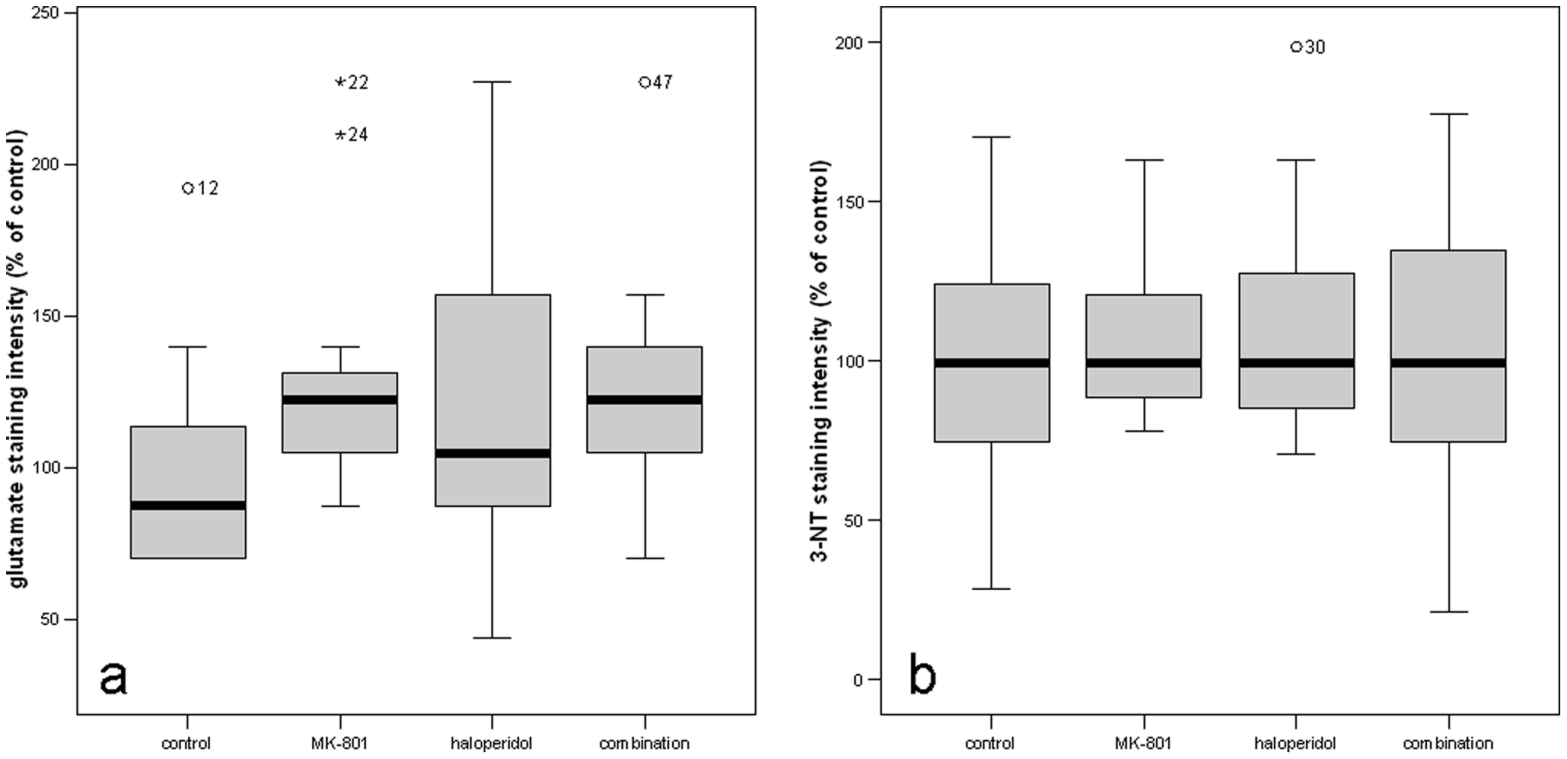
Treatment effects on glutamate (3a) and 3-nitrotyrosine (3b) staining intensity in the granular layer of the dentate gyrus. Tissue sections according to our anatomical definitions (→Materials and Methods) were selected from control animals (n = 12), MK-801 treated animals (n = 12), haloperidol treated animals (n = 11) and animals receiving a combination of both drugs (n = 12). For each animal, 8 individual sections were morphometrically evaluated. The granular layer dentate gyrus was manually outlined. The mean gray value was normalized to an area placed in the molecular layer of the dentate gyrus. For further analysis, the mean of these 8 measurements was calculated. The box plots represent the staining intensity relative to the mean staining intensity in the control group. * denotes statistical significance at p<0.05.

Intracellular glutamate staining was most prominent in the polymorphic zone (“CA4”) of the dentate gyrus (Fig. 2a). Ultrastructurally, the majority of these glutamate-rich cells were situated in the inner third of the polymorphic zone and seemed to represent “mossy cells”, which are characterized by a large soma with a triangular or multipolar shape and radially extending and bifurcating dendritic processes. Spines (“thorny excrescences”) could be observed at high magnification, corresponding to the termination of the mossy fiber axons. Singular intensely stained cells of mostly fusiform shape could be observed in the hilar region at the interface to the granular layer. Intense glutamate staining also became visible in the granular layer of the dentate gyrus. Interestingly, glutamate staining was enhanced by chronic treatment with MK-801 in this region (126.1+/−10.4% S.E.M of control; p = 0.036, Z = −2.09 Fig. 3a). Glutamate staining in other areas of the hippocampal formation or extrahippocampal brain regions (including prefrontal cortex, cingulate cortex, amygdala, thalamus or cerebellum) was not different between the treatment groups. Haloperidol alone did not alter the glutamate staining in any area under investigation. When applied as a “rescue therapy” after a 2 week course of MK-801 treatment, haloperidol did not attenuate the rise of intracellular glutamate levels in the dentate gyrus.

### Treatment effects on viability and glutamate metabolism in the hippocampal cell culture model

**Figure 4:**
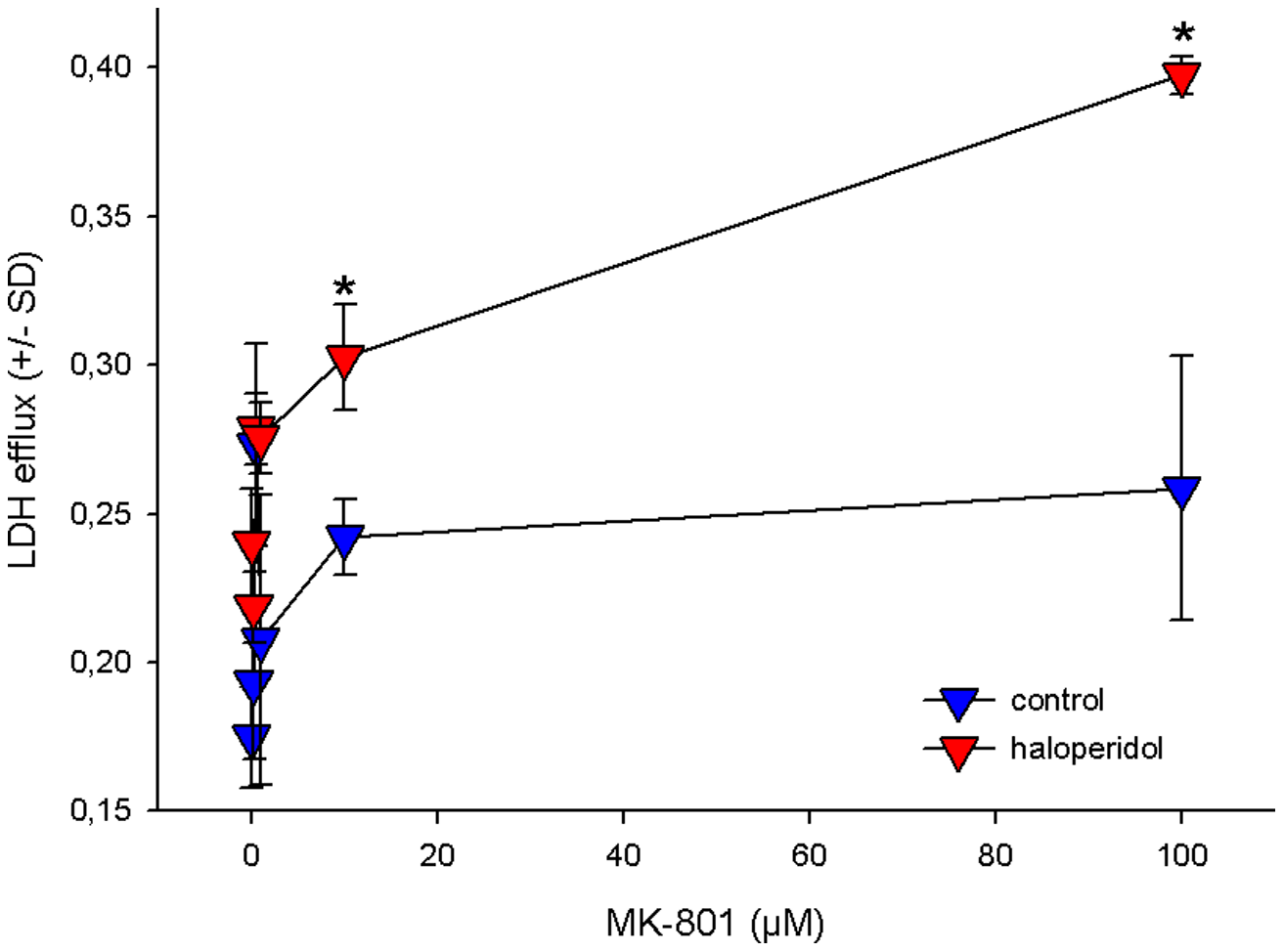
Effect of haloperidol on the cytotoxic action of MK-801. Hippocampal cells were pretreated with 5 µM haloperidol for 4 hours or left untreated. Afterwards, the cells were incubated for further 20 hours with different concentrations of MK-801, with haloperidol still being present. LDH efflux into the supernatant was chosen to assess cytotoxicity after 24 hours. To correct for cell mass, intracellular LDH was measured as a reference for each individual well after cell lysis. Data are expressed as ratio of extracellular LDH/total LDH and represent the mean +/− SD of 6 individual experiments each. * indicates statistical significance vs. untreated cells at p<0.001.

**Figure 5:**
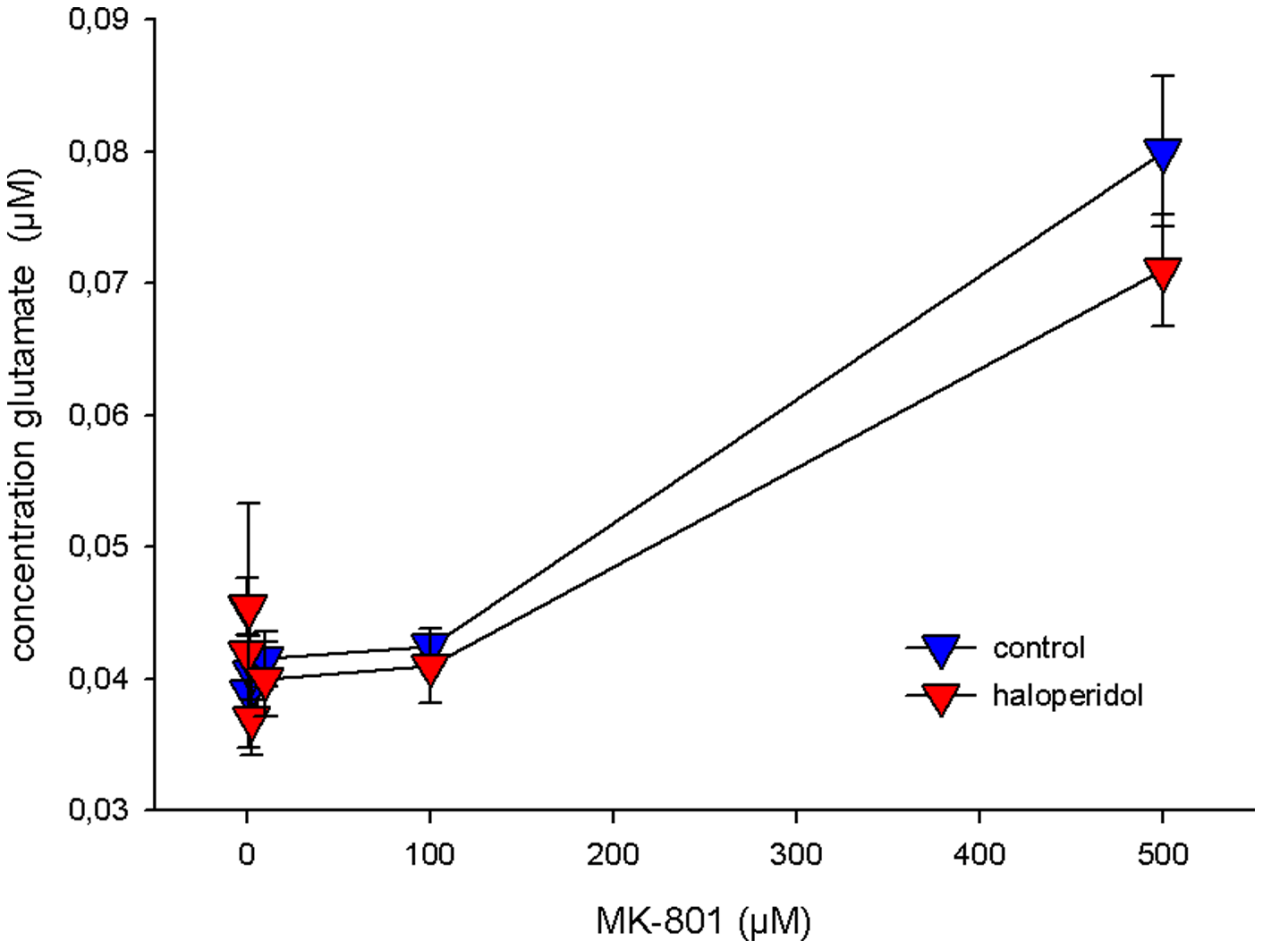
Effect of haloperidol on MK-801 induced glutamate efflux. Hippocampal cells were pretreated with 5 µM haloperidol for 4 hours and exposed to ascending concentrations of MK-801 for further 20 hours with haloperidol still being present. Glutamate was determined enzymatically in the culture supernatant. Data represent the mean +/− SD of 6 individual experiments each.

The toxicity of higher doses (>1 µM) of MK-801 became evident within 2–4 hours after application and proceeded throughout the entire incubation period (Fig. 4). Along with its dose-dependent toxicity, MK-801 enhanced the glutamate concentrations in the cell culture supernatant. In analogy to our observations in the animal model, haloperidol did not interfere with the changes in glutamate metabolism induced by MK-801 (Fig. 5). Unexpectedly, haloperidol even aggravated cytotoxicity determined by LDH efflux (Fig. 4). LDH levels were not correlated with glutamate levels (Spearman`s Rho correlation coefficient 0.13; p = 0.42) and –in contrast to the latter- showed a saturation kinetics. For this parameter, haloperidol shifted the maximal response to MK-801 from 0.242 AU (arbitrary units) to 0.351 AU, while the half-maximal effective dose remained largely unaffected (0.104 µM vs. 0.092 µM, respectively).

**Figure 6:**
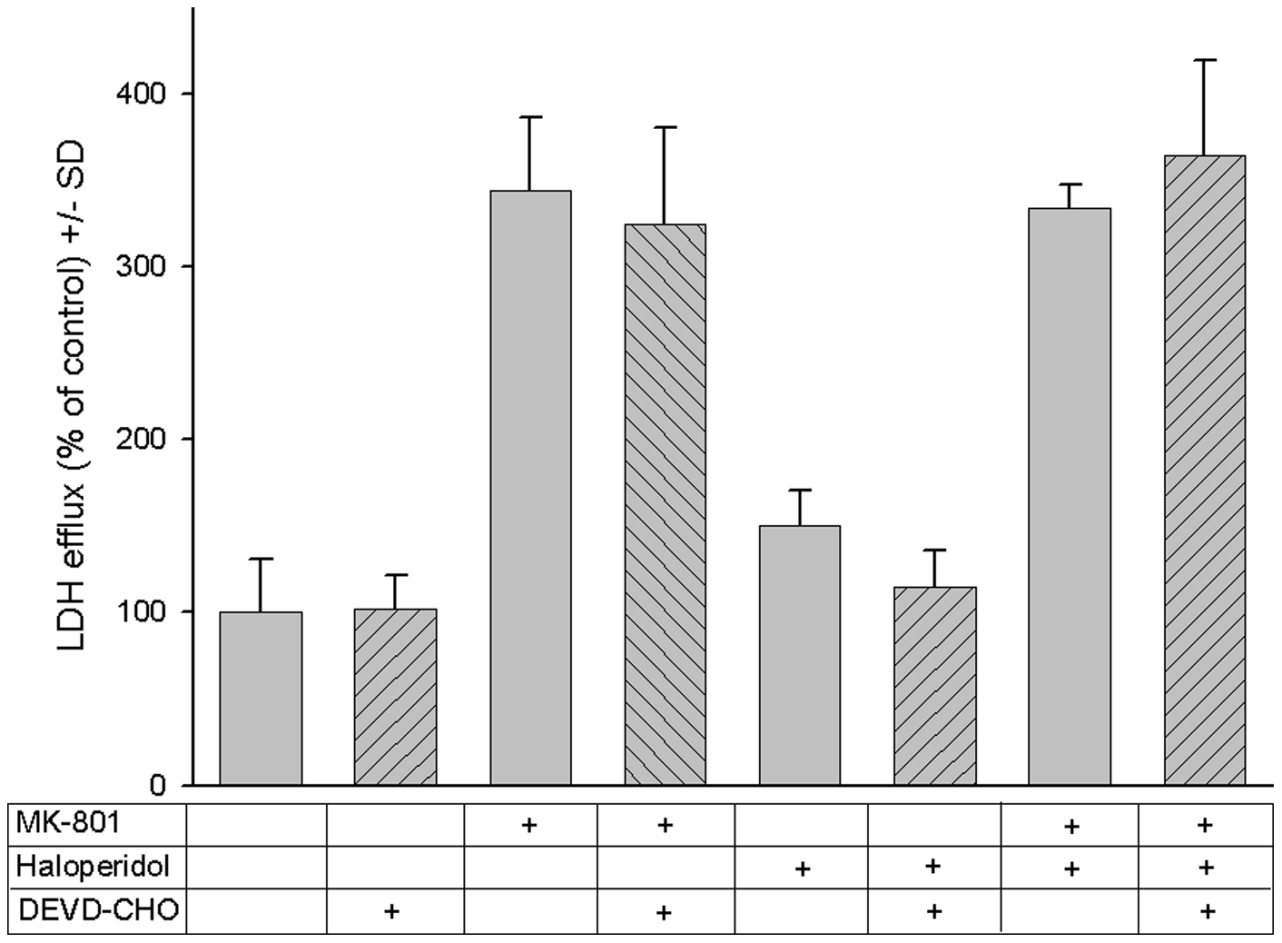
Role of apoptosis in MK-801 and haloperidol mediated neurotoxicity. Hippocampal cells were treated with MK-801 (7.5 µM), haloperidol (25 µM) and the caspase inhibitor DEVD-CHO (2.5 nM) or a combination of these agents. LDH efflux into the supernatant served as a measure of cytotoxicity after 24 hours. To correct for cell mass, intracellular LDH was determined as a reference for each individual well after cell lysis. The ratio of extracellular LDH/total LDH was calculated and data were expressed as% of cells left totally untreated (first bar). Each bar represents the mean +/− SD of 12 individual experiments. Under none of the treatment conditions DEVD-CHO yielded a statistically significant effect on cytotoxicity.

Apoptosis seemed to play a minor role in MK-801 and/or haloperidol mediated cytotoxicity, as the response was unaltered when the cell cultures were preincubated with the caspase-3 inhibitor peptide DEVD-CHO before addition of MK-801, haloperidol or a combination of both (Fig. 6).

### Assessment of oxidative stress in the animal model

3-nitrotyrosine (3-NT) immunostaining was chosen as a biomarker of cumulative ROI-induced nitrosylation of tyrosine residues. The staining pattern showed a similar topographical distribution as seen in glutamate stainings (Fig. 2b) with the highest abundance of intensely 3-NT positive cells in the dentate gyrus. Areas with low cellularity, as the hilar region or the stratum oriens of the hippocampus were relatively free from 3-NT staining and thus served as internal background controls. A linear regression analysis did not reveal a direct correlation between glutamate and 3-NT staining intensity (Spearman`s Rho correlation coefficient 0.11; p = 0.49). Moreover, in the inter-group comparison none of the treatments had any impact on the levels of oxidative stress in any brain region under investigation. Specifically, MK-801 did not affect the 3-NT levels in the dentate gyrus (Fig. 3b). The same was the case for haloperidol which has been reported to elicit oxidative stress in literature [29].

### Treatment effects on the formation of reactive oxygen intermediates in the cell culture model

**Figure 7:**
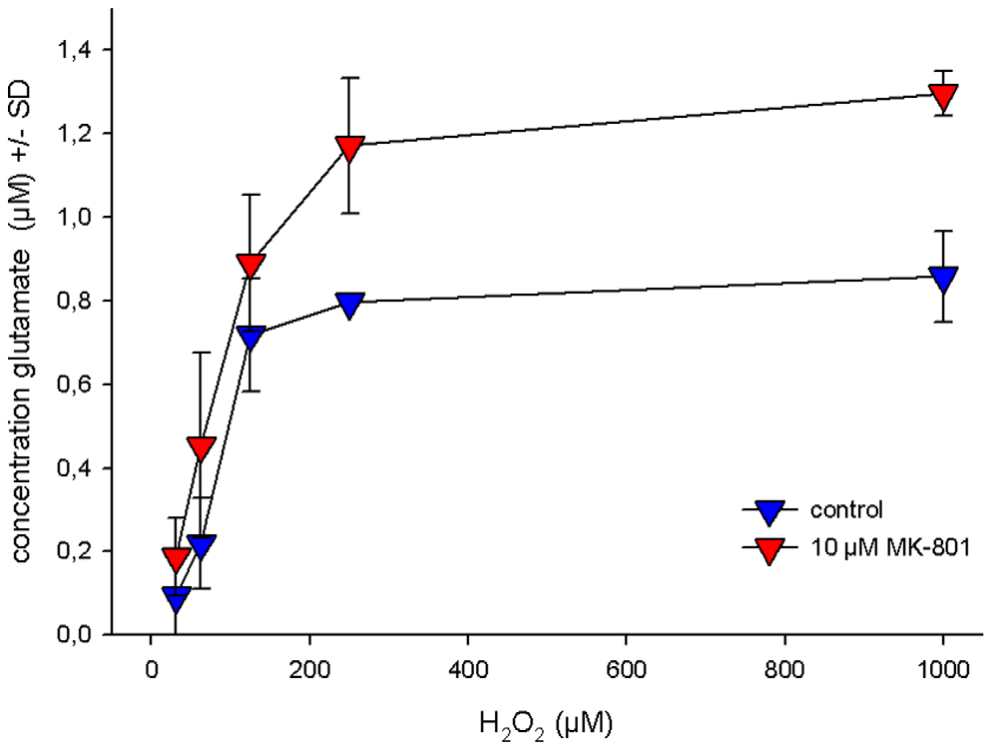
Effect of MK-801 on H_2_O_2_-induced glutamate efflux. Hippocampal cells pretreated with 10 µM MK-801 were exposed to ascending concentrations of H_2_O_2_ for 24 hours under normoxic conditions. Glutamate was determined enzymatically in the culture supernatant. Data represent the mean +/− SD of 6 individual experiments each. * denotes statistical significance vs. untreated cells at p<0.001.

Emulating conditions of mild oxidative stress by direct application of H_2_O_2_ to the cell culture revealed a positive feed-back loop between glutamate and generation of ROI even at amounts of H_2_O_2_ far to low to yield measurable toxicity (Fig. 7). Interestingly, antagonism of the NMDA receptor with MK-801, which we initially supposed to mitigate this response, even potentiated H_2_O_2_ induced glutamate spillover. Generation of superoxide anions (O_2_^.−^) in response to different agents was followed in real-time by determination of the lucigenin-enhanced chemiluminescence in suspensions of hippocampal cells. Here the peak basal O_2_^.−^ generation was transiently enhanced by 10 µm MK-801 (449+/−75% of baseline) as well as by haloperidol (440+/−45% of baseline) and remained elevated for the following minutes while the O_2_^.−^ levels in response to the drug combination were only slightly enhanced (163+/−71% of baseline). After administration of 100 µM glutamate, control cells responded with a 127+/−34% rise of O_2_^.−^ levels. This response was potentiated by MK-801 (217+/−63%) and haloperidol (255+/−28%). The same applied to the combination of both drugs (237+/−81%).

## Discussion

Employing an animal model of schizophrenia based on chronic low-dose application of the NMDA receptor antagonist MK-801 in a vulnerable developmental period, we have previously reported molecular, cellular, functional, and behavioral abnormalities which support the theory of NMDA receptor hypofunction in schizophrenia [15], [30]. Here we deliver further evidence that this model shares remarkable metabolic parallels with genuine schizophrenia, which could be further characterized in supplementary *in vitro* experiments. Beyond that, we hypothesized that the disinhibition of pyramidal cells resulting from impaired control by inhibitory GABAergic interneurons may enhance glutamate release and aberrant production of ROI. To address this question, we investigated these parameters in the abovementioned animal model and extended our experiments to hippocampal cell cultures. Immunohistochemically, we were able to demonstrate enhanced levels of intracellular glutamate staining in the hippocampal formation after chronic application of MK-801.

Our cell culture experiments revealed a similar response with an elevation of glutamate levels following MK-801 exposure even at low micromolar levels, which unexpectedly were not directly related to cytotoxicity. For the *in vitro* experiments it would have been desirable to be able to quantify the brain concentration range of non-protein bound MK-801 after chronical i.p. application. Unfortunately, no reliable pharmacokinetic data for MK-801 in rodents are found in literature, thus we decided to cover a broad concentration range. However, from experiments with [3H] labeled MK-801, showing that a single i.p. application of 0.2 mg/kg results in brain levels of 428 nM [31], we roughly estimated, that a repeated administration of 0.02 mg/kg should lead to brain tissue levels in the high nanomolar range.

Our cell culture data are in good agreement with previous reports having demonstrated an altered ratio of glutamate vs. glutamine in *post mortem* tissue and cerebrospinal fluid [24], [25]. Furthermore *in vivo* 1^H^-proton magnetic resonance spectroscopy studies have revealed that ketamine administration in healthy volunteers enhances cingular glutamine release [32]. In drug naive subjects at high genetic risk for schizophrenia this marker was also enhanced [33].

Taking into consideration the elevated glutamate levels observed in our study, we reasoned, that such imbalances of glutamate in the cellular microenvironment, may be directly associated to oxidative stress. An excessive generation of ROI is the core mediator of cellular malfunction or death. Interestingly, in recent studies experimental evidence for a direct link between the redox imbalance and the development of parvalbumin expressing interneurons has been delivered [34], an interneuron subtype which has shown to be reduced in schizophrenia [35], [36].

Indeed, our hippocampal cell culture experiments revealed a pro-oxidant effect of MK-801, no matter whether hippocampal cells were investigated under basal conditions or after glutamate exposure. While it is a well-known phenomenon that glutamate release promotes enhanced ROI formation, we could additionally demonstrate that ROI *vice versa* can enhance extracellular glutamate release. Thus, MK-801 may initiate and maintain a feedback circuit, which would ultimately result in neurodegeneration. The presence of redox-sensitive sites at the NMDA-R [37]–[40] or direct disturbance of presynaptic and astrocytic glutamate uptake by an altered redox status may be possible explanations for this phenomenon.

Nevertheless, it was not possible to deliver direct evidence for enhanced oxidative stress with 3-NT in our animal model.

Taken together, it should be stated that our experiments deliver partially conflicting results when comparing the in vivo and in vitro data, suggesting that interpretation of cell culture experiments has certain intrinsic limitations, which may be primarily explained by the absence of glial cells: the complicated interplay between neurons and astrocytes with respect to glutamate metabolism has not received enough attention to date. Therefore co-culture experiments would be desirable. Specifically, the glutamate-glutamine cycle, which maintains a direct metabolic link between both cell populations thereby establishing a homeostasis between extra- and intracellular glutamate levels would deserve more attention [41]. Focusing on the glial-neuronal interaction in a recent paper, Kondziella et al. were able to deliver evidence for a disrupted glutamate-glutamine cycling [42], potentially mediated by the inhibitory effect of MK-801 on astrocytic NMDA-receptors [43], which ultimately results in enhanced glutamate levels. Furthermore, an impaired glial-neuronal interaction and a concomitantly disrupted glutamate-glutamine metabolism could be demonstrated by magnetic resonance spectroscopy analysis of temporal lobe tissue from rats subjected to chronic MK-801 treatment [44]. As glial cells represent below 1% of the cell population in our hippocampal cell cultures, the additional level of complexity suggested by these studies only applies to our in vivo experiments and may underline the value of our strategy combining in vivo and in vitro data.

An intriguing aspect from our *in vitro* analysis is the massive potentiation of MK-801 toxicity by haloperidol and the discrepancy between glutamate and LDH after haloperidol co-administration, which may indicate that the neuroprotective effect of haloperidol is not directly mediated by effects on glutamate metabolism. Actually, glutamate may be elevated through a yet unknown mechanism at the pre- or postsynaptic sites of glutamatergic neurotransmission, unrelated to cytotoxic damage.

The interpretation of antipsychotic effects on glutamate-mediated neurotransmission remains controversial. Accumulating data suggest that cortical and subcortical glutamate release seems to be tuned by dopaminergic circuitries [45], [46]. Haloperidol has a significant affinity for NMDA receptors, thus inducing functional receptor alterations, which in turn might exacerbate glutamate transmission deficits apart from a direct elevation of glutamate levels as shown in microdialysis experiments [47], [48]. Data from primary striatal culture and animal experiments demonstrate that the intraneuronal signal transduction pathway activated by haloperidol, the cAMP pathway, leads to phosphorylation of the NR1 subtype of the NMDA receptor [49] and may even increase the number of NMDA receptors in different cortical regions [50]. Besides, direct NMDA receptor modulation by haloperidol, a further level of reciprocal interaction between the glutamatergic and dopaminergic transmitter systems is provided by a D2/D4 receptor mediated tyrosine kinase transactivation, which elicits a cascade in turn leading to a depression of NMDA-R mediated synaptic transmission [51]. Such actions may reinforce the disturbance of glutamatergic neurotransmission induced by MK-801. Apart from their interaction with NMDA-receptors, antipsychotics are hypothesized to enhance striatal glutamatergic neurotransmission by blocking presynaptic dopamine receptors, thereby causing neuronal damage by oxidative stress [52]. Interestingly, symptoms of tardive dyskinesia provoked by long-term haloperidol therapy correlated positively with markers of oxidative stress [53]. The positive interaction between NMDA- receptor antagonism and dopamine-receptor antagonism is also reflected in EEG data showing that the modest EEG slowing induced by haloperidol and MK-801 individually is massively potentiated when the drugs are combined [54]. The same is true for the phenomenon of disturbed prepulse inhibition (PPI), which haloperidol fails to normalize [55]. These complex interactions certainly require further investigation, specifically considering that amelioration of positive and negative symptoms by antipsychotic drugs may be related to actions on different receptor systems or intracellular pathways. As cognitive impairment was prevailing in our model, our data may deliver further evidence that conventional antipsychotics may be inadequate in targeting schizophrenia-related cognitive impairment. Further support for this interpretation can be derived from our histological data, which lack evidence for an amelioration of MK-801 induced glutamate dysregulation by haloperidol.

Although the alterations induced by chronic NMDA antagonism are unlikely to represent schizophrenia in its entire complexity, we were able to deliver evidence that this approach represents a valid model for at least some of the core deficits occurring in this condition. We were able to provide some further support to the notion that local imbalances of glutamatergic neurotransmission caused by disordered NMDA receptor function may be implicated in some of the schizoisomorphic functional and neuropathological alterations in our animal model. The contribution of oxidative stress may represent another attractive explanation, namely for some of the neurodegenerative features. However, at the present stage our data do not indicate that oxidative stress seems to play a major role. This may have direct consequences for the development of new therapeutic strategies for schizophrenia. Our data disclose no rationale for the application of antioxidants, which have repeatedly been promoted as promising new therapeutic agents [56]–[59]. Our results may rather indicate that drugs directly interfering with glutamate metabolism, such as AMPA modulators may constitute a superior pharmacological approach [60], [61]. Certainly, this issue will require further investigations, which will be essentially dependent on the establishment and characterization of valid models for psychosis.
